# Outcomes after extracorporeal membrane oxygenation following the norwood procedure: a systematic review and meta-analysis

**DOI:** 10.1186/s13019-026-04232-4

**Published:** 2026-05-23

**Authors:** Abdullah Almehandi, Adel Altarkait, Yahya Ali, Mohammad Alhajri, Mohammed Alenezi, Lucas Diniz, Abdulrahman O. Al-Naseem, Hamood Al Kindi, Saif Awlad thani

**Affiliations:** 1 Department of Cardiovascular Science, University College London, 118 E Ferry Rd, London, E14 3NW UK; 2 General Surgery, Sheikh Jaber Al-Ahmad Al-Sabah Hospital, Kuwait City, Kuwait; 3 Faculty of Life Sciences and Medicine, King’s College London, London, UK; 4 School of Medicine, University of Leeds, Leeds, UK; 5 Department of Surgery, Al-Adan Hospital, Kuwait City, Kuwait; 6 Department of Medical Sciences, Federal Fluminense University, Niterói, Brazil; 7 Department of Surgery, McGill University, Montréal, Canada; 8 Department of Cardiothoracic Surgery, Sultan Qaboos University Hospital, Sib, Oman; 9 Pediatric Intensive Care Unit, The Royal Hospital, Muscat, Oman

**Keywords:** ECMO: Extracorporeal membrane oxygenation, CPB: Cardiopulmonary bypass, ICU: Intensive care unit, ECLS: Extracorporeal life support

## Abstract

**Background:**

Survival following the Norwood procedure has improved substantially, but outcomes for the high-risk subgroup requiring postoperative extracorporeal membrane oxygenation (ECMO) remain unclear. We explored short- and intermediate-term outcomes in these neonates.

**Main Body:**

We systematically searched PubMed, EMBASE, Cochrane Central and Web of Science (final search: 25 February 2025). Comparative studies reporting outcomes for patients with versus without postoperative ECMO were included. Primary outcomes were short-term mortality, secondary outcomes included stages-2-and-3 palliation completion, operative times, recovery parameters, and complications. Statistical analysis used RevMan 5.4 to calculate pooled risk ratio (RR), odds ratios (OR) or mean differences (MD) with 95% confidence intervals employing random-effects models; heterogeneity was assessed using I² statistics and Cochran’s Q test.

Eight studies (2,612 patients: 15% with ECMO) were included. In-hospital mortality was five times greater in the ECMO group (RR = 5.53, CI = 4.41–6.93, *p* = 0.0003), though survival to Glenn and Fontan completion were comparable. Cardiopulmonary bypass duration was significantly longer in the ECMO group (MD = 28.07 min, CI = 18.19–37.95, *p* = 0.0004). Postoperative ICU recovery was significantly prolonged for ECMO patients (MD = 10.56 days, CI = 1.28–19.83, *p* = 0.03), though hospital recovery was similar. Postoperative atrioventricular valve regurgitation, Norwood reoperation, delayed chest closure, unplanned reoperation or death/transplantation were comparable. Risk of bias (ROBINS-I) was formally assessed.

**Conclusion:**

Our analysis supports that ECMO use post-Norwood procedure identifies a high-risk subgroup with significantly higher early-mortality and resource utilisation. However, this association must be interpreted cautiously due to heterogenous ECMO indications. Future efforts should focus on identifying modifiable risk factors to improve outcomes.

**Supplementary Information:**

The online version contains supplementary material available at 10.1186/s13019-026-04232-4.

## Introduction

Hypoplastic left heart syndrome (HLHS) and related single ventricle malformations represent some of the most severe forms of congenital heart disease, characterised by underdevelopment of the left-sided cardiac structures. Surgical management requires a series of staged palliative operations, beginning with the Norwood stage I procedure in the neonatal period, followed by the bidirectional cavopulmonary connection (BCPC) and finally the Fontan procedure [1]. Despite advances in surgical techniques and perioperative care, the Norwood procedure remains associated with significant perioperative morbidity and mortality, with reported in-hospital mortality rates ranging from 10% to 20% [2–4].

Extracorporeal membrane oxygenation (ECMO) is frequently used as a rescue therapy for neonates who develop severe low cardiac output syndrome, refractory hypoxaemia, or failure to wean from cardiopulmonary bypass (CPB) after Norwood palliation [2, 5]. Although ECMO can be life-saving by providing temporary cardiopulmonary support, its use in this high-risk population is often associated with significant complications, prolonged intensive care, and a high risk of mortality [2, 5, 6]. Consequently, the survival benefit of ECMO in this setting remains controversial, with previous studies reporting variable survival rates and limited evidence comparing outcomes between patients who require ECMO and those who do not [2, 5–7].

Understanding whether ECMO provides a survival advantage or simply reflects severe perioperative instability and thus leads to a worse prognosis is critical for perioperative decision-making, risk stratification, and parental counselling in this patient cohort [5, 6]. While individual institutional reports have described outcomes for patients supported with ECMO after the Norwood procedure, no comprehensive systematic review and meta-analysis have directly compared survival and mortality between patients receiving ECMO and those who managed without it.

Therefore, our meta-analysis aims to assess whether there is a difference in mortality and survival through palliation stages, including perioperative outcomes between neonates who undergo ECMO and those who are not following the Norwood procedure for HLHS and other forms of single ventricle disease. By clarifying the impact of ECMO use on early survival and recovery, this study seeks to inform future clinical practice and guide discussions with families facing this high-risk procedure.

## Materials and methods

This systematic review and meta-analysis were conducted in accordance with the Preferred Reporting Items for Systematic Reviews and Meta-Analyses (PRISMA) guidelines [8].

### Eligibility criteria

Inclusion in this meta-analysis was restricted to studies that met al.l the following eligibility criteria: [1] randomised trials or non-randomised cohorts; [2] including cohorts that underwent ECMO post-Norwood; [3] enrolling patients who underwent Norwood procedure for single ventricle disease. In addition, studies were included only if they were written in [4] English language, and if they reported any of the clinical outcomes of interest. We excluded studies including [1] case report or case series methodology; [2] no control group; [3] patients undergoing pre-operative ECMO [4] reported outcomes not being stratified by ECMO use in Norwood repair; [5] patients undergoing Hybrid stage I palliation; [6] single ventricle patients undergoing a different stage palliation.

We recognised that, among the included studies, the cohorts from Friedland-Little et al. (2014) and Friedland-Little et al. (2017) both included infants who required ECMO support after a Norwood operation at the University of Michigan between January 2001 and December 2010. To avoid potential duplication of patient data, we ensured that no forest plot pooled data from both studies simultaneously.

### Search strategy and data extraction

Two authors independently searched for English-language articles in PubMed, Web of Science, Cochrane Central Register of Controlled Trials and EMBASE from inception to January 2025 with the following search terms: The following search terms were used in various combinations with Boolean operators: ‘single ventricle’, ‘Norwood’, ‘HLHS’, ‘Norwood Procedure’, ‘ECMO’, ‘ECLS’, ‘Mechanical Circulatory Support’, ‘VA-ECMO’, ‘paediatric ECMO’, ‘ECPR’ (Supplementary Table 1). The references from all included studies, previous systematic reviews and meta-analyses were also searched manually for any additional studies. Two authors independently extracted the data following predefined search criteria and quality assessment.

### Quality assessment

Risk of bias was assessed for non-randomised studies via the ROBINS-I [9]. These studies were assessed against seven domains including: confounding, classification of interventions, selection of participants, deviation from the intended intervention, missing data, outcome measurement and selection of the reported result. Risk assessment included low (green), moderate (yellow) and serious risk (red) (Supplementary Table 2).

### Outcomes

Primary outcome was short-term mortality (defined as pre-discharge or 30 days post-discharge). Secondary Outcomes included: Glenn and Fontan completion, interstage mortality or transplantation, cardiopulmonary bypass time (CPB) (minutes), cross clamp time (minutes), hypothermic circulatory arrest time (HCA) (minutes), hospital and intensive care unit (ICU) length of stay (days), and postoperative complications including Norwood reoperation, delayed chest closure, moderate to severe atrioventricular valve regurgitation (AVVR) and unplanned reoperation.

Unplanned reoperations were defined according to indications reported in the primary studies, including shunt-related complications, residual lesions (such as shunt stenosis or residual arch obstruction requiring repair), and re-exploration for bleeding. Cardiac catheterization interventions were not included in the pooled unplanned reoperation outcome. Part of our analysis plan was to stratify the analyses based on ECMO indication (failure to wean, E-CPR, hypoxemia) given its potential impact on outcomes. Unfortunately, outcome data from pooled studies was not stratified by indication to allow appropriate quantitative subgroup analysis. Therefore, all analyses reflect aggregate outcomes for ECMO use irrespective of indication.

### Statistical analysis

Odds ratios (OR) and 95% confidence intervals (CI) were calculated for each outcome. An OR greater than 1, or an MD greater than zero, corresponds to a greater frequency of the studied outcome. Continuous variables were analyzed using mean difference (MD) and 95% confidence intervals (95% CI). Inherent clinical heterogeneity between the studies was balanced via the implementation of a random effects model (Hartung-Knapp-Sidik-Jonkman). Results were displayed in forest plots. Alpha error was set to 0.05. Between-study statistical heterogeneity was assessed with the Cochran Q statistic and by estimating I^2^. High heterogeneity was confirmed with a significance level of *p* < 0.10 and I^2^ of at least 50% or more. All statistical analyses were performed using Review Manager 5.3.

In parallel, linear regression analyses were conducted for interstage death or transplantation, survival to Glenn, unplanned reoperation, cross-clamp time (minutes), ICU and hospital length of stay, comparing them with publication year. The regression models were weighted by population size, included 95% confidence intervals for the fitted curves, and reported R² values, where values close to 1 indicate a strong association and values close to 0 indicate no association.

## Results

### Study characteristics

**Fig. 1:**
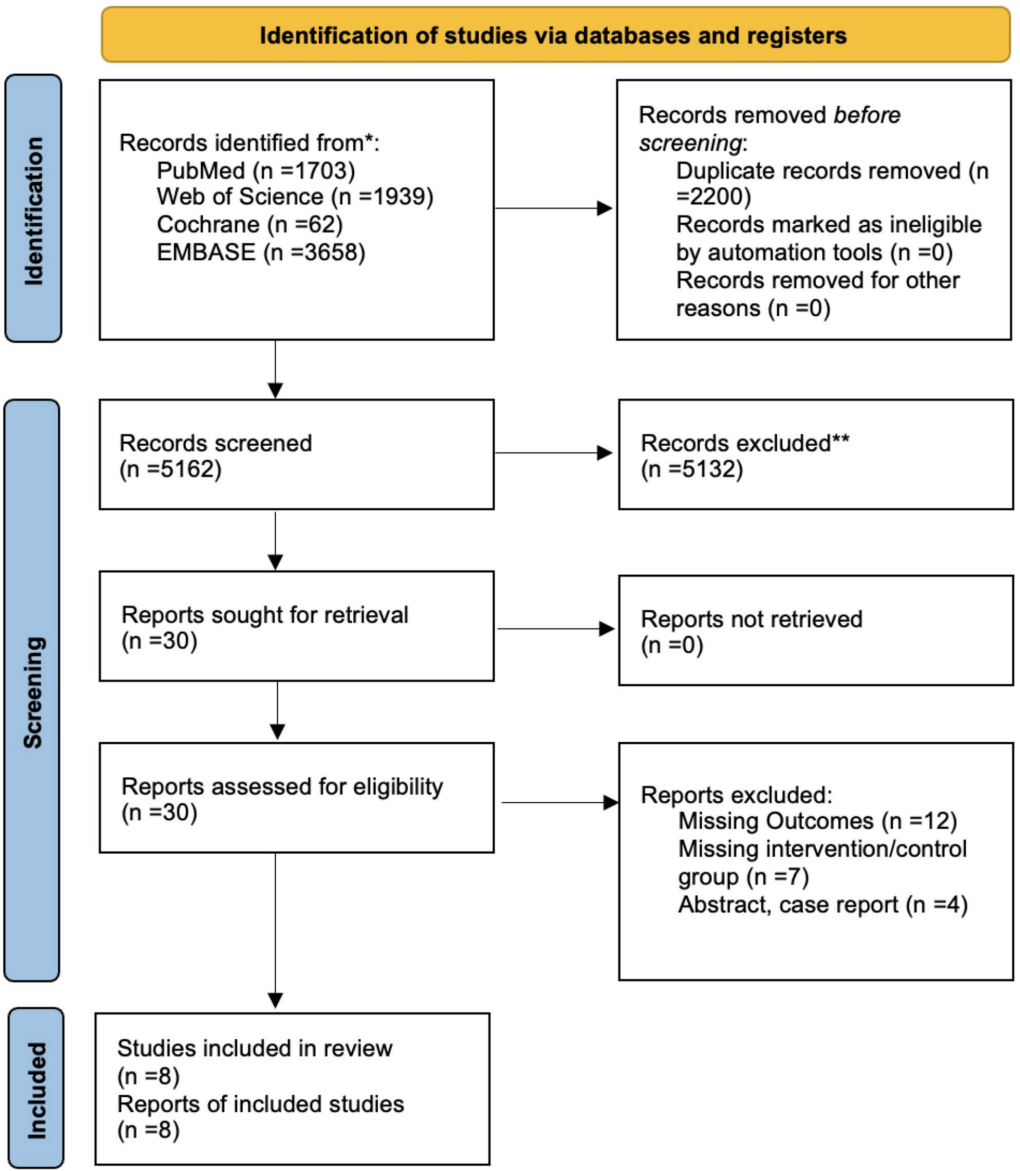
PRISMA flow diagram for study screening

A total of 5,162 studies were screened from the systematic search and 30 were sought for retrieval, of which 8 met the criteria for inclusion in the final analysis. Figure 1 shows the PRISMA flowchart for study selection. Included studies were published between 2009 and 2025, and all 8 included studies were observational trials [2, 10–16].

Table 1 shows the details of the included studies. A total of 2,612 patients were included in the final analysis. The number of patients in each study ranged from 31 to 931.

**Table 1:** Summary of included studies

Author	Year of Publication	Country	Number of Patients	Study Design	Mean Follow-up (years)	Reported Outcomes
Alsoufi	2015	USA	284	Single Centre Retrospective Cohort Study	4.5	CPB time (min), CVVH, neurologic complications, and bleeding
Beshish	2024	USA	269	Single Centre Retrospective Cohort Study	N/A	Systemic ventricular function, CPB time (minutes), cross-clamp time (minutes), HCA time (minutes), post-Norwood FiO₂ on arrival to the CICU, post-Norwood catheter-based intervention, duration of mechanical ventilation, length of stay (days), operative mortality, and progression to stage 2 palliation were evaluated
De Jesus-Brugman	2020	USA	85	Single Centre Retrospective Cohort Study	3.23	Indexed shunt diameter (mm/m²), CPB duration (minutes), aortic cross-clamp duration (minutes), regional cerebral perfusion, open sternum (including open sternum with open skin and open sternum duration in days), cardiac arrest (in the operating room, ICU, or cardiology ward), ICU stay, hospital stay, neurologic injury, and operative mortality were assessed
Fernandez	2017	USA	931	Multicenter Retrospective Cohort Study	N/A	CPB time, aortic cross-clamp time, circulatory arrest time, thoracic and non-thoracic surgeries after stage I palliation, postoperative catheter intervention, postoperative rhythm abnormalities, postoperative complications (excluding ECMO), age at discharge (days), weight at discharge (kg), oxygen saturation at discharge, and death or transplant during the interstage period were assessed
Friedland-Little	2013	USA	255	Single Center Case-control Study	N/A	CPB time (min), ACC time (min), HCA time (min), Postoperative, Highest postoperative lactate within 48 h of surgery, Maximum postoperative VIS within 48 h of surgery
Friedland-Little	2017	USA	31	Single Centre Prospective Cohort Study	N/A	Number of operations, postoperative length of stay, cardiopulmonary bypass (CPB) time, transplantations, Fontan completion, Pediatric Quality of Life Inventory (PedsQL) scores, and comparisons of caregiver- and patient-reported PedsQL scores with the general population
Mayr	2022	Germany	257	Single Centre Retrospective Cohort Study	1.4	Cardiopulmonary bypass (CPB) time (min), aortic cross-clamp (AXC) time (min), hypothermic circulatory arrest (HCA) time (min), duration of intubation, intensive care unit (ICU) length of stay (LOS), and hospital LOS
Sengupta	2022	USA	500	Single Centre Retrospective Cohort Study	N/A	Outcomes included cross-clamp time, circulatory arrest time, antegrade cerebral perfusion, more than one cardiopulmonary bypass run (for or unrelated to residual lesions), Blalock–Taussig shunt, chest left open, residual lesions involving the proximal arch, distal arch, coronary arteries or Stansel anastomosis, atrial septum, neoaortic valve, Blalock–Taussig or Sano shunt, and branch pulmonary arteries, as well as any intraoperative residual lesion, age, mechanical ventilation, and cardiopulmonary bypass time

CPB: cardiopulmonary bypass; ACC: aortic cross-clamp; AXC: aortic cross-clamp; HCA: hypothermic circulatory arrest; CICU: cardiac intensive care unit; ICU: intensive care unit; LOS: length of stay; VIS: vasoactive-inotrope score; CVVH: continuous veno-venous hemofiltration; PedsQL: Pediatric Quality of Life Inventory; FiO₂: fraction of inspired oxygen; ECMO: extracorporeal membrane oxygenation; min: minutes; kg: kilograms; mm/m²: millimeters per square meter

### Patient characteristics

Table 2 summarises the demographic data of the patient population in each study. The proportion of patients that underwent ECMO post-Norwood procedure with varied between 7.1% and 39%. The mean age of patients ranged from 4 to 9 days old, while the proportion of female patients varied between 8% and 45%. The mean weight at the time of surgery ranged from 3.1 kg to 3.3 kg.

**Table 2:** Demographic data of the patient population

Author (*n* = sample size)	Number of Patients (%)		Age (days), Median (IQR/range), Mean ± SD		Female (%)		Weight, kg Median (IQR/range), Mean ± SD		ECPR (%)	Prematurity (%)		Extracardiac anomaly (%)		Cardiac diagnosis (%)		HLHS (%)	
	E	NE	E	NE	E	NE	E	NE		E	NE	E	NE	E	NE	E	NE
** Alsoufi et al. 2015**[10] **(***n* **= 284)**	38 (15)	246 (87)	6.0 (4–7)	5.0 (3–7)	17 (45)	93 (38)	-	-	22 (58)	5 (13)	29 (12)	5 (13)	31 (13)	9 (24)	56 (23)	29 (76)	190 (77)
** Beshish et al. 2024**[11] **(***n*** = 264)**	65 (24)	204 (76)	6.0 (4–7)	5.0 (4–7)	25 (39)	77 (38)	3.2 (3–4)	3.2 (3–4)	20 (36)	-	-	14 (22)	47 (23)	15 (23)	44 (23)	50 (77)	152 (75)
** De Jesus-Brugman et al. 2020**[12] **(***n* **= 85)**	25 (29)	60 (71)	9 (8–12)	9 (8–12)	-	-	3.1 (3–4)	3.2 (3–4)	21 (84)	-	-	-	-	-	-	-	-
** Fernandez et al. 2017**[13] **(***n*** = 931)**	66 (7)	865 (93)	8.9 ± 10.6	8.4 ± 17	27 (41)	317 (37)	3.37 ± 0.6	3.26 ± 0.54	-	-	-	9 (14)	87 (10)	57 (86)	778 (90)	66 (100)	865 (100)
** Friedland-Little et al. 2013** **(***n*** = 255)**	64 (25)	191 (75)	6 (5–8)	7 (5–8)	23 (36)	67 (35)	3.1 ± 0.6	3.3 ± 0.5	-	11 (17)	18 (9)	16 (25)	29 (15)	1 (2)	21 (11)	63 (99)	170 (89)
** Friedland-Little et al. 2017**[15] **(***n* **= 31)**	12 (39)	19 (61)	5.5 (3–10)	6 (2–11)	1 (8)	6 (32)	3.3 (1.8–3.9)	3.3 (2.1–4.2)	-	1 (8.3)	1 (5.3)	2 (16.7)	3 (15.8)	2 (17)	7 (37)	12 (100)	15 (79)
** Mayr et al. 2022**[2] **(***n*** = 257)**	41 (16)	216 (84)	8 (7–13)	9 (7–12)	18 (41)	69 (32)	3.2 (3–4)	3.2 (3–4)	4 (10)	0 (0)	11 (5)	8 (18)	23 (11)	18 (44)	83 (38)	33 (81)	157 (73)
**Sengupta et al. 2022** **(***n***= 500)**	78 (16)	422 (84)	5 (3–6)	4 (3–6)	29 (37)	152 (36)	3.1 (3–4)	3.2 (3–4)	6 (8)	13 (17)	13 (17)	20 (26)	82 (19)	-	-	-	-

E: ECMO; NE: NO ECMO; ECMO: Extracorporeal Membrane Oxygenation; IQR: interquartile range; SD: standard deviation; ECPR: extracorporeal cardiopulmonary resuscitation; HLHS: hypoplastic left heart syndrome

Various single ventricle anomalies were observed in the patient population. Cardiac anomalies (ranging from 1.6% to 44%) to were generally greater in proportion compared to extracardiac anomalies (ranging from 10% to 25.6%). The prevalence of HLHS ranged from 74.5% to 100%, the ranges of HLHS defect incidences included: 6.2% to 46% mitral atresia/aortic atresia, 1.5% to 11.6% mitral atresia/aortic stenosis, 17.1% to 32% mitral stenosis/aortic atresia and 15.7% to 26% mitral stenosis/aortic stenosis.

### Quality and publication bias assessment

In terms of ROBINS-I assessment (Supplementary Fig. 2), an overall serious risk of bias was identified in 2 studies (Fernandez 2017 and Friedland-Little 2017), primarily due to confounding and patient selection respectively. Most remaining studies (Alsoufi 2014, Beshish 2024, De Jesus-Brugman 2020, Friedland-Little 2014 and Sengupta 2022. Reasons for moderate bias included risk of confounding, selection of participants, missing data and selection of the reported results.

On ROBINS-I assessment, Fernandez et al. was judged to have a serious risk of bias due to confounding, as although multiple clinically relevant prognostic variables were collected, the analysis relied on unadjusted univariable comparisons without adjustment for baseline illness severity, procedural heterogeneity, or center-level variation. This limits causal inference, as observed associations may reflect differences in case-mix rather than an independent effect of exposure; therefore, findings from this study should be interpreted as associative rather than causal.

Friedland-Little et al. demonstrated serious risk of bias in participant selection because the analysed cohort was restricted to surviving patients whose families consented to participation, which may limit representativeness of the eligible population and reduce confidence that reported outcomes reflect true group differences.

Additional sources of bias included missing outcome data in Friedland-Little et al. and Friedland-Little et al., which may have introduced uncertainty where follow-up was incomplete or unequal between groups, reducing confidence in long-term estimates. Selective reporting bias was also identified in Alsoufi et al., Beshish et al., and De Jesus-Brugman et al., where multiple analyses were performed without a clearly prespecified analysis plan and variable selection partly relied on statistical significance thresholds, increasing the possibility of selective emphasis on significant findings and potentially exaggerating observed associations.

In terms of assessment of publication bias, no definitive evidence of publication bias can be demonstrated, but publication bias cannot be excluded due to the limited number of available primary studies to include in our pooled analysis. Egger’s regression test demonstrated statistically significant funnel plot asymmetry (intercept = − 2.91, 95% CI − 3.67 to − 2.15, t = − 7.487, *p* = 0.017), suggesting the presence of small-study effects and potential publication bias (Supplementary Fig. 1).

### Pooled analysis of all studies

***Primary outcomes***

**Fig. 2:**
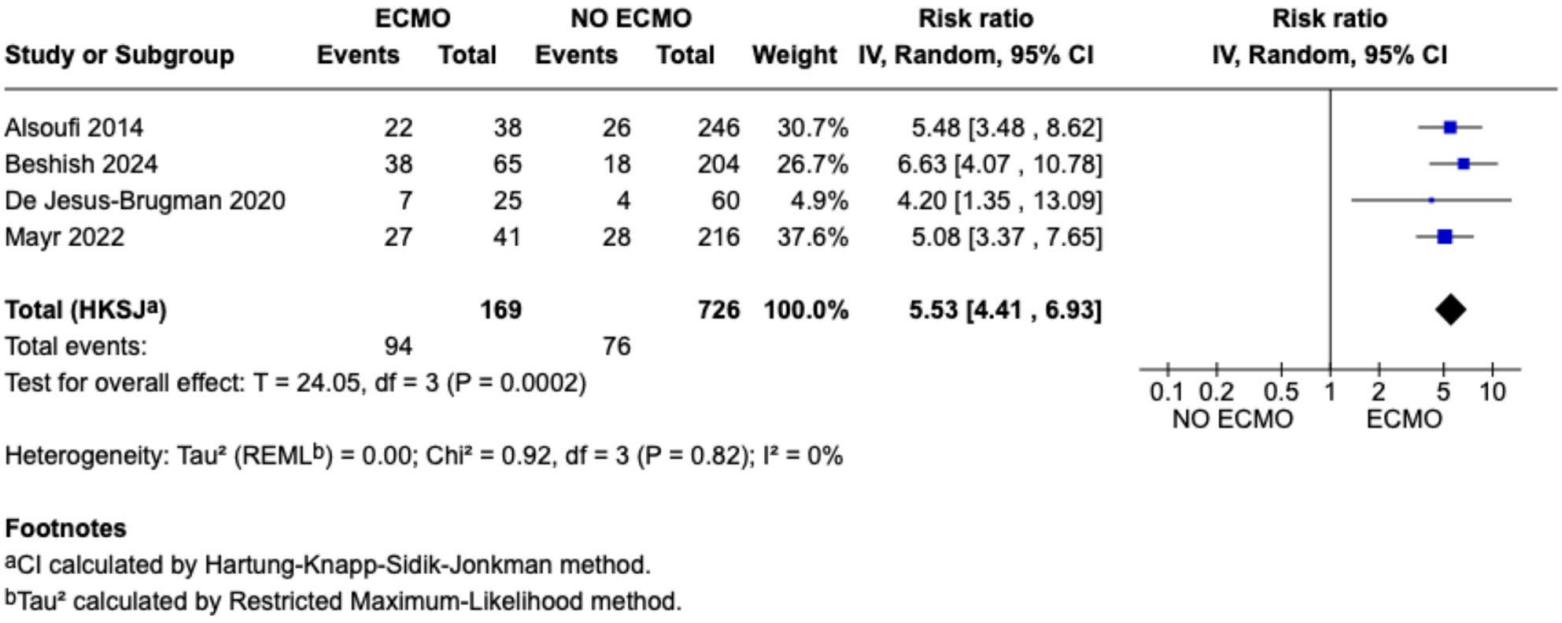
Forest plot for Short-term mortality. ECMO= Extracorporeal Membrane Oxygenation, MD= Mean Difference, OR= odds ratio, RR= risk ratio, CI= confidence interval

Table 3 outlines the detailed results of the meta-analysis. Figure 2 shows the forest plot of short-term mortality, which was reported by 4 studies and included a total of 895 patients. The short-term mortality of patients with ECMO was significant greater in patients who underwent the Norwood procedure, compared to non-ECMO patients post-Norwood (RR = 5.53, CI = 4.30 to 7.11, *P* < 0.00001) with low heterogeneity observed (I2 = 0%, *p* = 0.82).

**Table 3:** Outcomes summary

Outcome	Number of Studies	Number of Patients	Effect Estimate (95% CI, *p*-value)
**Short-term Mortality**	4	895	RR = 5.53, CI = 4.30 to 7.11, *p* < 0.00001
**Glenn Completion**	3	611	OR = 0.15, CI = 0.02 to 1.06, *p* = 0.05
**Fontan Completion**	3	497	OR = 0.18, CI = 0.01 to 2.98, *p* = 0.12
**CPB time (mins)**	8	1,927	MD = 28.07, CI = 18.19 to 37.95, *p* = 0.0004
**Cross-clamp time (mins)**	7	1927	MD = 2.91, CI= −0.53 to 6.34, *p* = 0.08
**HCA time (mins)**	5	1558	MD = 187, CI= −1.28 to 5.02, *p* = 0.17
**Hospital LOS (days)**	4	642	MD = 15.84, CI= −3.59 to 35.26, *p* = 0.08
**ICU LOS (days)**	3	611	MD = 10.56, CI = 1.28 to 19.83, *p* = 0.03
**Unplanned Reoperation**	3	308	OR = 5.69, CI = 1.03 to 31.44, *p* = 0.05
**Interstage Mortality/Transplantation**	4	1821	OR = 2.95, CI = 0.54 to 16.24, *p* = 0.14
**Atrioventricular Valve Regurgitation**	4	1,281	OR = 2.03, CI = 0.76 to 5.43, *p* = 0.11

RR: risk ratio; MD: mean difference; OR: odds ratio; CI: confidence interval; CPB: cardiopulmonary bypass; HCA: hypothermic circulatory arrest; LOS: length of stay; ICU: intensive care unit

***Secondary outcomes***

**Fig. 3:**
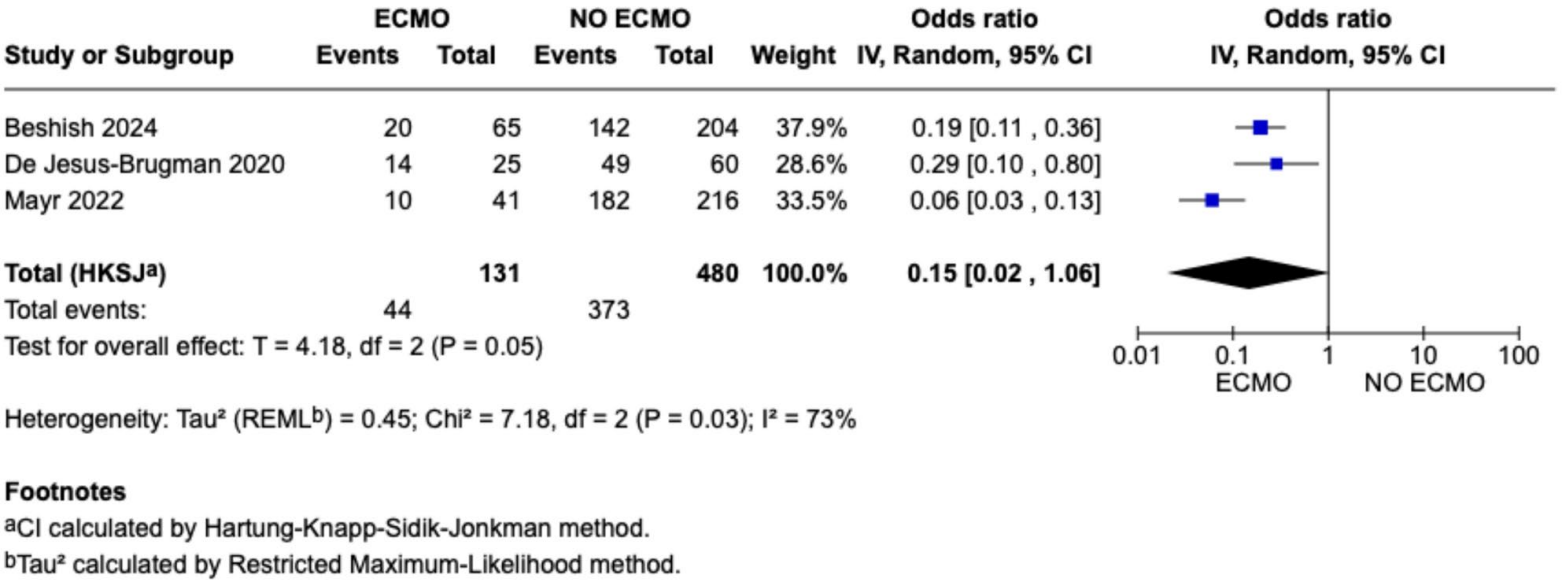
Forest plot for Glenn Completion. ECMO= Extracorporeal Membrane Oxygenation, MD= Mean Difference, OR= odds ratio, RR= risk ratio, CI= confidence interval

Figure 3 shows the forest plot for the number of patients who reached Glenn surgery, which was reported by 3 studies and included a total of 611 patients. The pooled analysis shows this quantity was comparable between patients who underwent postoperative ECMO compared to the group without ECMO (OR = 0.15, CI = 0.02 to 1.06, *P* = 0.05) with high heterogeneity observed (I2 = 73%, *p* = 0.03). Leave-on-out analysis successfully reduced the heterogeneity, though pooled analysis remains insignificant (OR = 0.21, CI = 0.02 to 1.84, *P* = 0.07), (I2 = 0%, *p* = 0.52) (Supplementary Fig. 1).

**Fig. 4:**
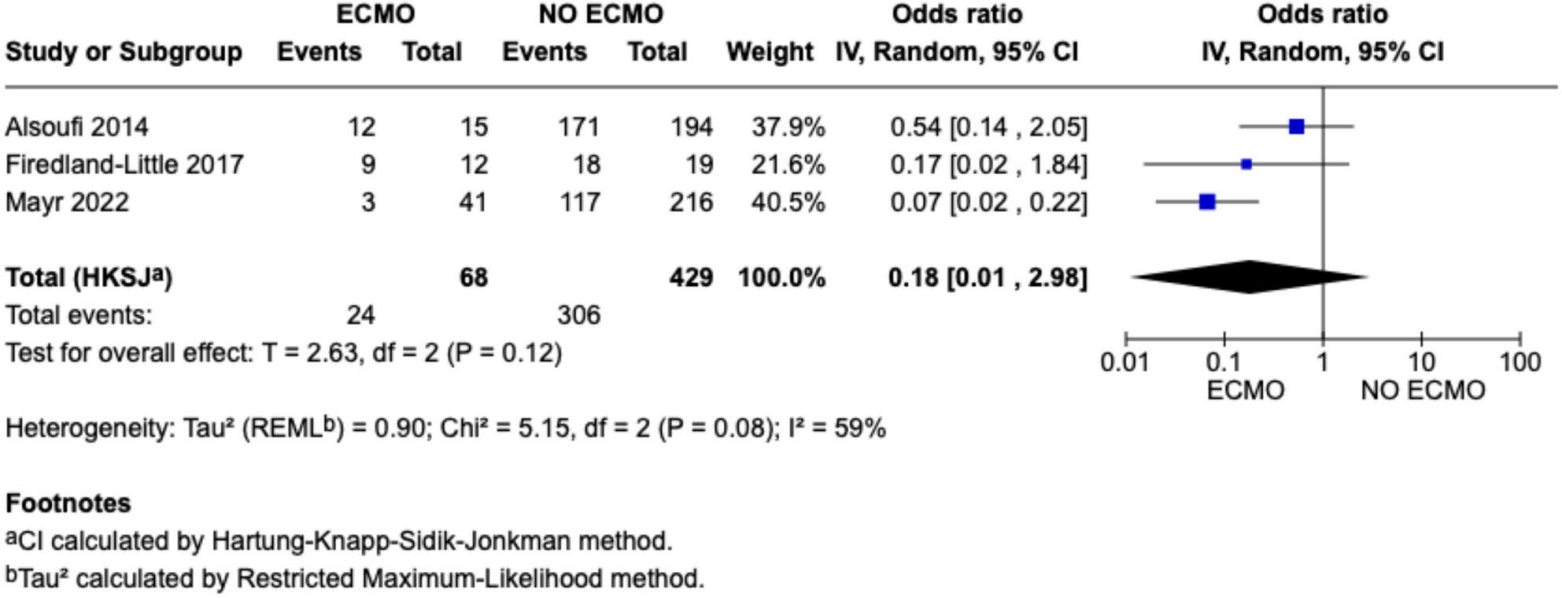
Forest plot for Fontan Completion. ECMO= Extracorporeal Membrane Oxygenation, MD= Mean Difference, OR= odds ratio, RR= risk ratio, CI= confidence interval

Figure 4 shows the forest plot for Fontan completion, which was reported by 3 studies and included a total of 497 patients. Fontan completion by patients who underwent post-Norwood ECMO was comparable compared to the group without ECMO post-Norwood (OR = 0.18, CI = 0.01 to 2.98, *P* = 0.12) with moderate heterogeneity observed (I2 = 59%, *p* = 0.08).

**Fig. 5:**
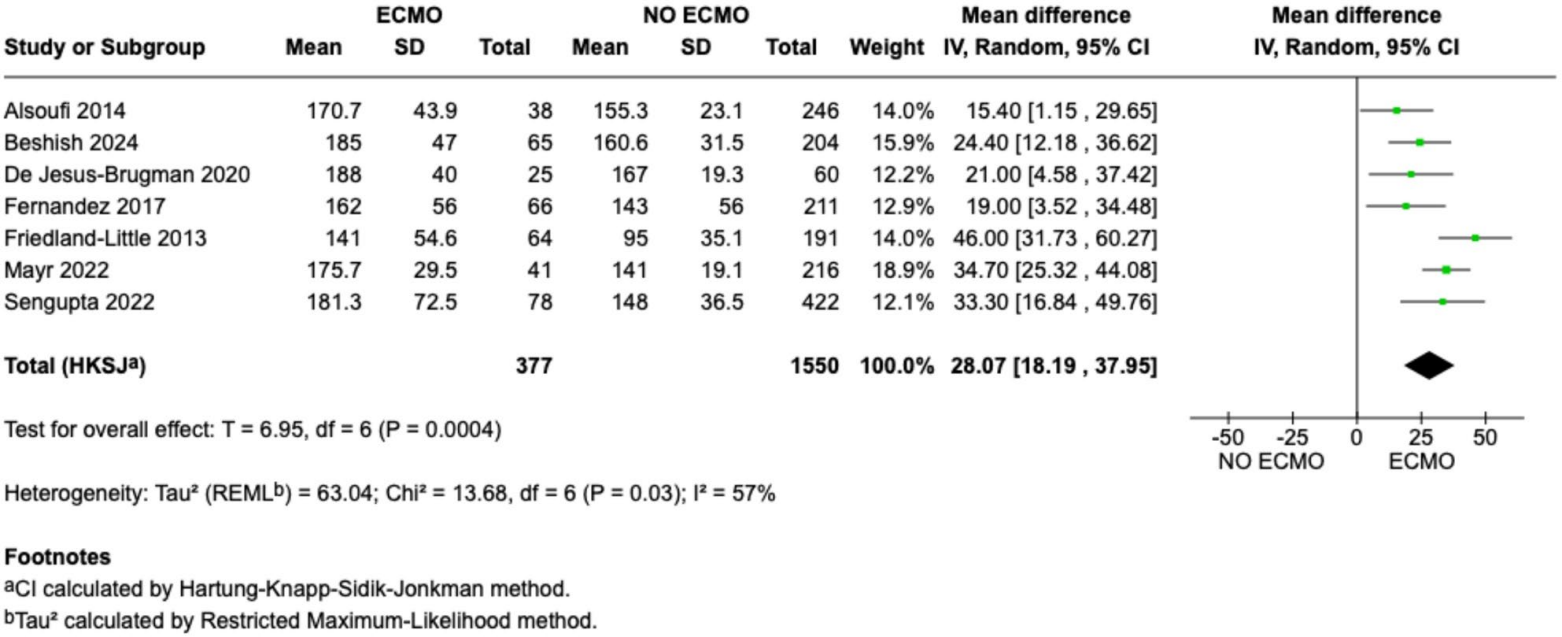
Forest plot for CPB time (minutes). ECMO= Extracorporeal Membrane Oxygenation, MD= Mean Difference, OR= odds ratio, RR= risk ratio, CI= confidence interval

Figure 5 shows the forest plot for the duration of cardiopulmonary bypass (minutes) from 8 studies, which included a total of 1,927 patients. When compared to the ECMO group, CPB time was significantly shorter in the group non-ECMO group (MD = 28.07, CI = 18.19 to 37.95, *p* = 0.0004) with moderate heterogeneity observed (I2 = 57%, *p* = 0.03).

**Fig. 6:**
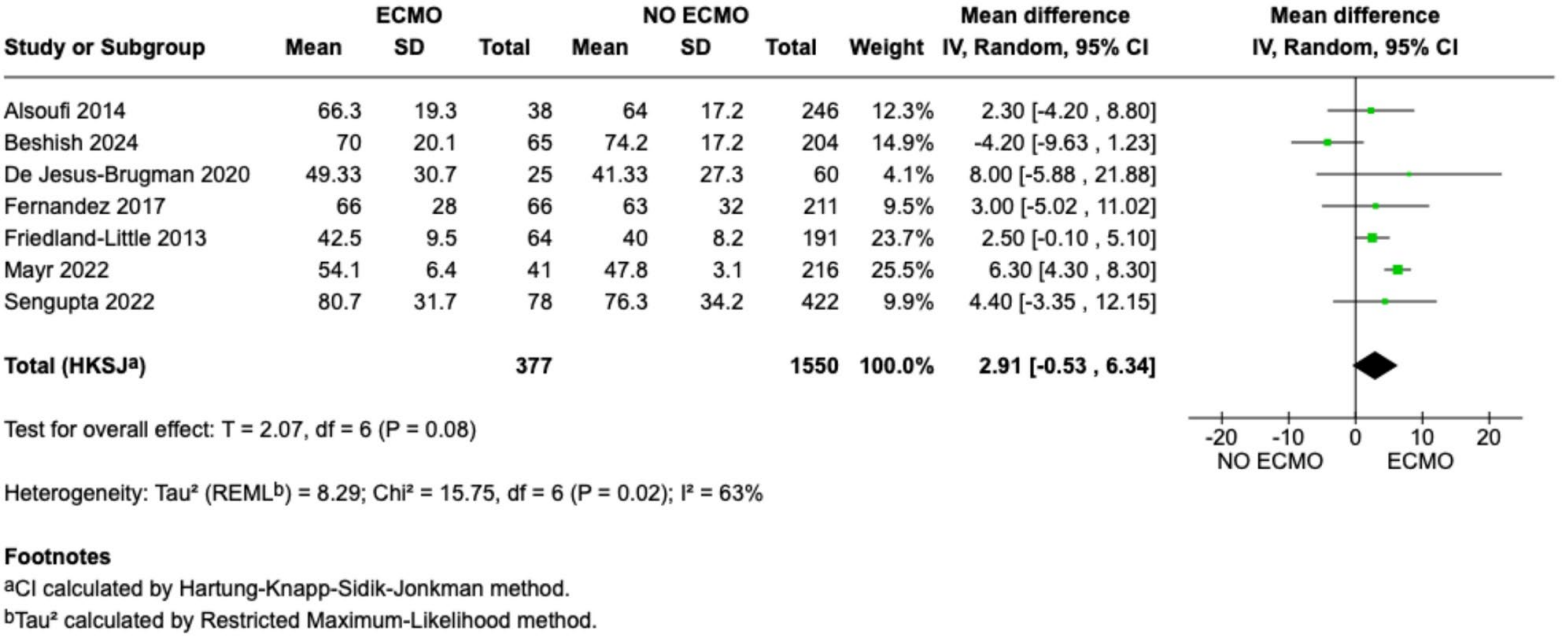
Forest plot for Cross-clamp time (minutes). ECMO= Extracorporeal Membrane Oxygenation, MD= Mean Difference, OR= odds ratio, RR= risk ratio, CI= confidence interval

Figure 6 shows the forest plot for cross-clamp time (mins) from 7 studies, which included a total of 1,927 patients. When compared to the group without ECMO, patients undergoing ECMO following Norwood repair had comparable cross-clamp times (MD = 2.91, CI= −0.53 to 6.34, *p* = 0.08) with a high level of heterogeneity observed (I2 = 63%, *p* = 0.02). Leave-on-out analysis successfully reduced the heterogeneity, though pooled analysis remains insignificant (MD = 0.21, CI = 0.02 to 1.84, *P* = 0.07), (I2 = 0%, *p* = 0.52) (Supplementary Fig. 2).

**Fig. 7:**
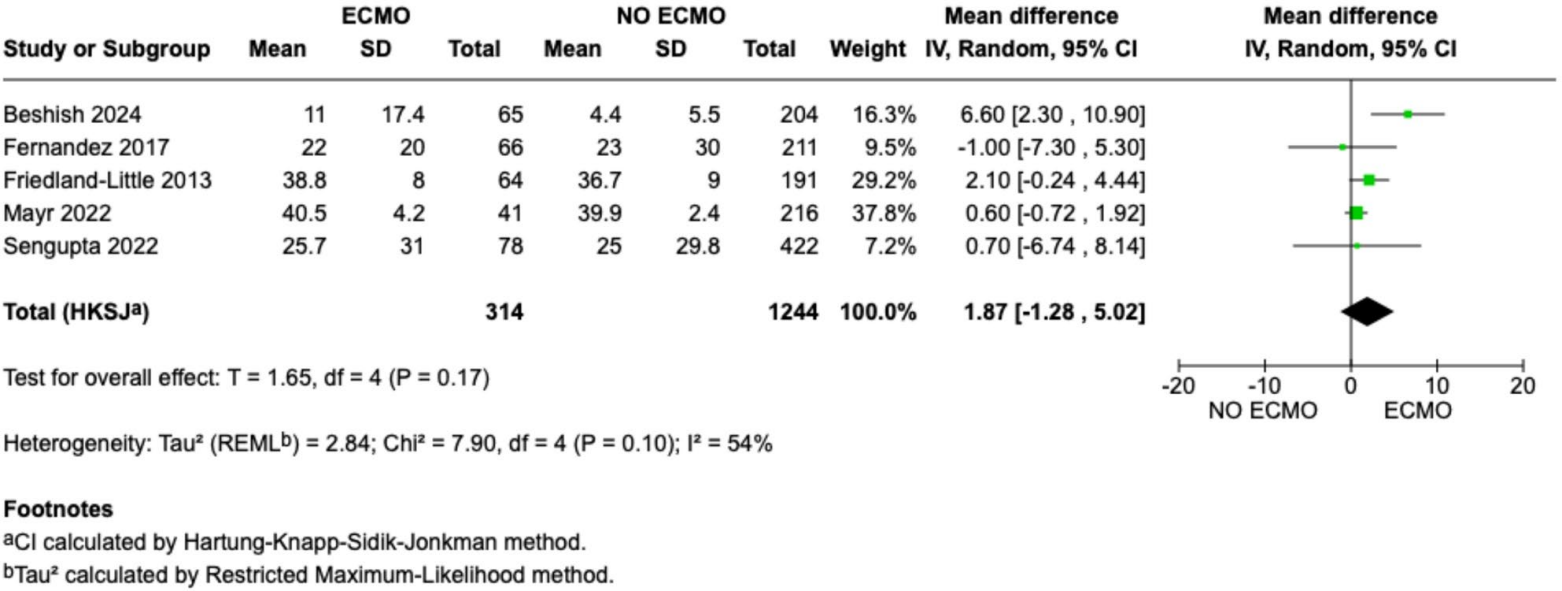
Forest plot for Hypothermic Circulatory Arrest (minutes). ECMO= Extracorporeal Membrane Oxygenation, MD= Mean Difference, OR= odds ratio, RR= risk ratio, CI= confidence interval

Figure 7 shows the forest plot for HCA time (minutes) from 5 studies, which included a total of 1,558 patients. When compared to the ECMO group, the duration of HCA in the group without ECMO was comparable (MD = 187, CI= −1.28 to 5.02, *p* = 0.17) with moderate heterogeneity observed (I2 = 54%, *p* = 0.10).

**Fig. 8:**
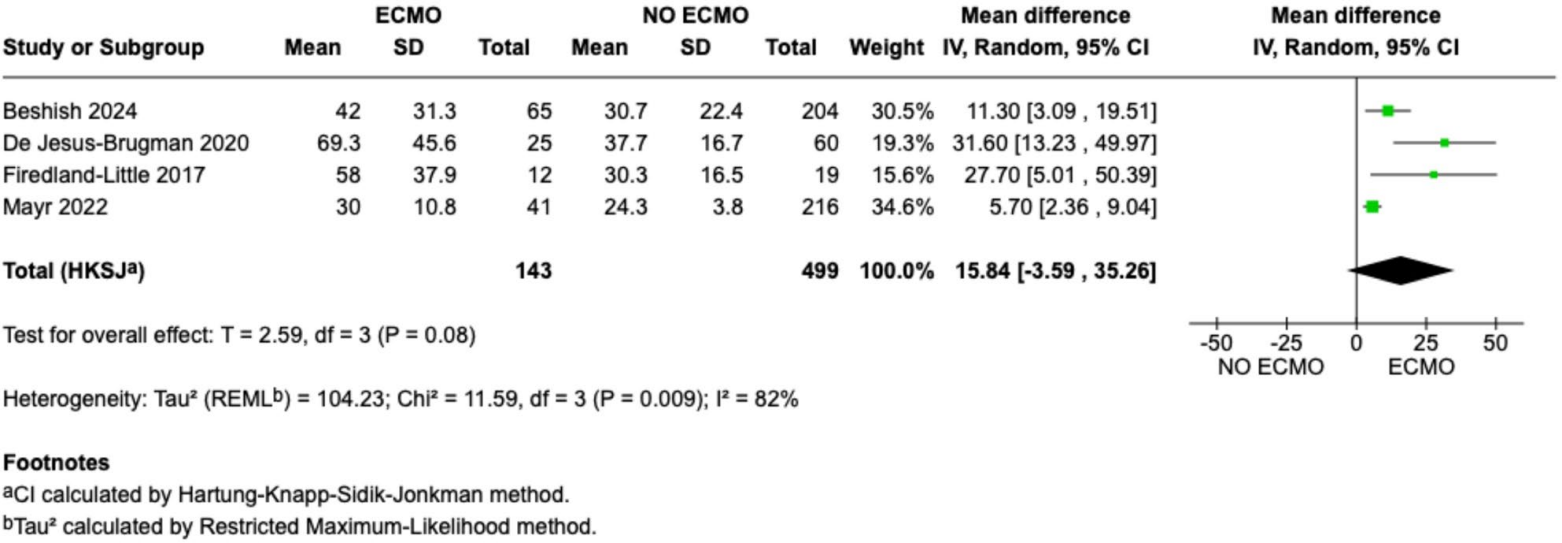
Forest plot for Hospital Length of Stay (days). ECMO= Extracorporeal Membrane Oxygenation, MD= Mean Difference, OR= odds ratio, RR= risk ratio, CI= confidence interval

Figure 8 shows the forest plot for hospital length of stay (days) from 4 studies, which included a total of 642 patients. When compared to the ECMO group, the duration of hospital stay (days) was similar in the non-ECMO group (MD = 15.84, CI= −3.59 to 35.26, *p* = 0.08) with high heterogeneity observed (I2 = 82%, *p* = 0.009). Leave-on-out analysis failed to significantly reduce the heterogeneity, pooled analysis remains insignificant (MD = 9.65, CI= −9.33 to 28.64, *P* = 0.16), (I2 = 55%, *p* = 0.09) (Supplementary Fig. 3).

**Fig. 9:**
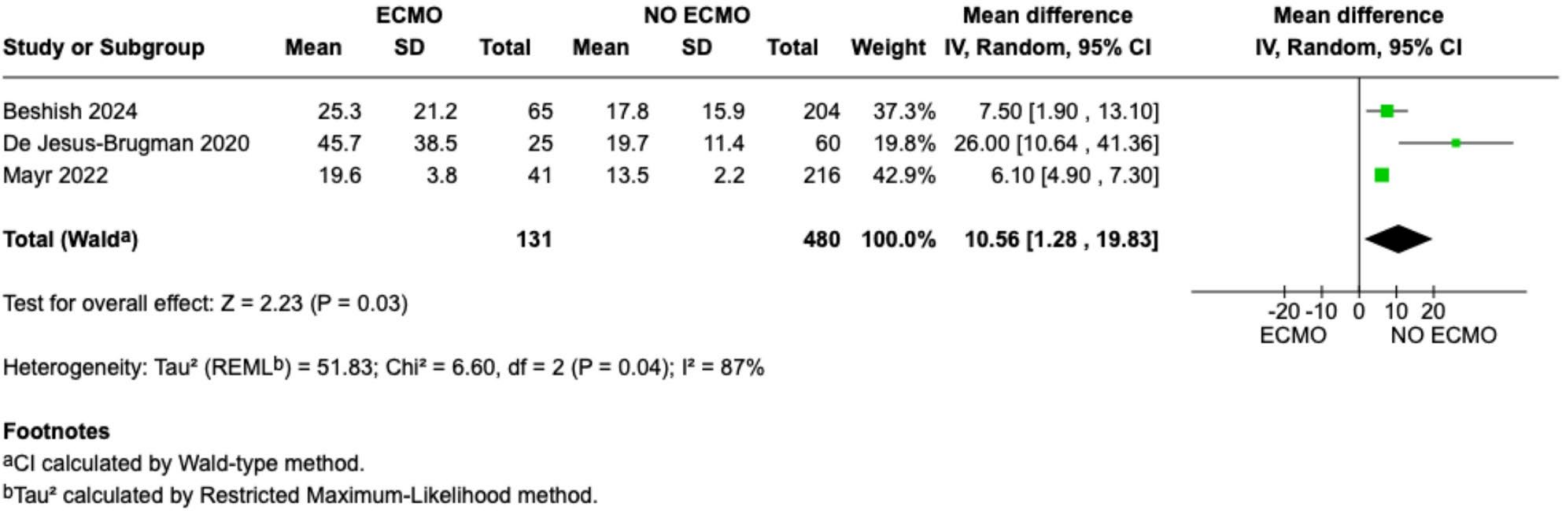
Forest plot for ICU Length of Stay (days). ECMO= Extracorporeal Membrane Oxygenation, MD= Mean Difference, OR= odds ratio, RR= risk ratio, CI= confidence interval

Figure 9 shows the forest plot for ICU length of stay (days) from 3 studies, which included a total of 611 patients. When compared to the ECMO group, the duration of ICU stay (days) was significantly shorter in the non-ECMO group (MD = 10.56, CI = 1.28 to 19.83, *p* = 0.03) with high heterogeneity observed (I2 = 87%, *p* = 0.04). Leave-on-out analysis successfully reduced the heterogeneity, though pooled analysis remains insignificant (MD = 6.16, CI = 4.99 to 7.33, *P* < 0.00001), (I2 = 0%, *p* = 0.63) (Supplementary Fig. 4).

Supplementary Fig. 5 shows the forest plot for unplanned reoperation from 4 studies, which included a total of 308 patients. The overall incidence of unplanned reoperation was comparable (OR = 5.69, CI = 1.03 to 31.44, *p* = 0.05). Heterogeneity was reported to be high (I2 = 81%, *p* = 0.0008). Leave-on-out analysis successfully reduced the heterogeneity and resulted in significant pooled analysis favouring less unplanned reoperation in the ECMO group post-Norwood (OR = 3.65, CI = 1.15 to 11.61, *P* = 0.04), (I2 = 0%, *p* = 0.43) (Supplementary Fig. 6).

Pooled data from 4 studies including a total of 1821 patients, highlighting the incidence of interstage death or transplantation is presented in Supplementary Fig. 7. Results show comparable incidence of interstage mortality or transplantation between the two cohorts (OR = 2.95, CI = 0.54 to 16.24, *p* = 0.14). Heterogeneity was reported to be high (I2 = 61%, *p* = 0.05). Leave-on-out analysis successfully reduced the heterogeneity, though pooled analysis remained incomparable (OR = 2.01, CI = 0.15 to 26.73, *P* = 0.37), (I2 = 20%, *p* = 0.17) (Supplementary Fig. 8).

Supplementary Fig. 9 shows the forest plot for postoperative AVVR, reported by 4 studies, which included a total of 1,281 patients. Pooled analysis shows comparable incidence of AVVR (OR = 2.03, CI = 0.76 to 5.43, *p* = 0.11) with moderate heterogeneity observed (I2 = 39%, *p* = 0.18).

### Meta regression

The meta-regression analysis comparing interstage death or transplantation, survival to Glenn and unplanned reoperation were all insignificant when compared to year of publication (coefficient = 0.31; *p* = 0.44), (coefficient = 0; *p* = 1) and (coefficient = 0.05; *p* = 0.77) (Supplementary Figs. 11–13) respectively. Similarly, our meta-regression analysis for cross-clamp time (minutes) and recovery duration in ICU and in hospital were insignificant (coefficient = 0.01; *p* = 0.81), (coefficient = 0.46; *p* = 0.52) and (coefficient = 0.34; *p* = 0.42) respectively (Supplementary Figs. 14–16).

## Discussion

Summary of findings

In this systematic review and meta-analysis of 8 studies and 2,612 patients, we compared the use of ECMO versus standard post-operative management in single ventricle disease patients undergoing Norwood procedure. The main findings from our pooled analysis include: [1] significantly greater incidence of short-term (in-hospital) mortality in patients who underwent ECMO initiation postoperatively; [2] comparable completion of the Glenn and Norwood procedures; [3] significantly longer CPB times (minutes) in the ECMO group, and although approaching significance, cross-clamp and HCA times (minutes) were both comparable; [4] a significantly longer ICU length of stay postoperatively in the ECMO group, though hospital length of stay was similar; and finally [5] comparable incidence of unplanned reoperations, interstage mortality/transplantation and atrioventricular valve regurgitation.

The requirement for ECMO following neonatal heart surgery is a well-established predictor of poor prognosis [5, 17]. This risk is profoundly amplified in the specific context of the Norwood procedure, which is widely recognised as one of the highest-risk procedures in congenital cardiac surgery [18]. The elevated short-term mortality observed in our cohort of patients receiving post-Norwood ECMO is corroborated by contemporary studies, which consistently report in-hospital mortality rates ranging from 50% to 70% for this critically ill population [4, 19, 20]. Evidence from literature has suggested ECMO support to be an independent predictor of 30-day and in-hospital mortality following the Norwood procedure [21].

In contrast, according to other studies, a more principal independent predictor of short-term mortality following Norwood repair is the need for cardiopulmonary resuscitation (CPR) in the perioperative period, rather than ECMO itself. This distinction is significant, because a cardiac arrest requiring CPR may be a marker of profound underlying physiological instability [12]. In particular, the tenuous hemodynamic state of Norwood circulation, such an event could signify a severe disruption in the critical balance between coronary perfusion and myocardial oxygen demand [22]. It has therefore been suggested that mortality may be more directly linked to the non-modifiable patient factors precipitating cardiac arrest than to the potentially modifiable morbidities associated with ECMO support itself [12].

The significant institutional variability in reported outcomes for post-Norwood ECMO underscore the critical need to identify precise indications for its initiation, particularly those distinguishing it from catastrophic cardiac arrest [23]. Common triggers, such as low cardiac output syndrome, cardiovascular collapse, persistent hypoxemia, and refractory arrhythmias, are not equivalent in their prognostic implications. For example, published data highlights previous observations that cannulation for hypoxaemia or shunt thrombosis is associated with a markedly better chance of survival to discharge than support initiated secondary to cardiovascular collapse [24]. This means that, owing to the multitude of ECMO indications, our finding of significantly higher incidence of short-term mortality in the ECMO cohort post-Norwood procedure should be interpreted with caution.

The aforementioned heterogeneity in clinical indications, which serves as a moving target for comparative analysis, is an important consideration to make when interpreting the inter-institutional outcome differences [25]. A second, significant factor is institutional expertise. This is evidenced by the clear contrast between large registry data, which report survival rates approaching 31%, and individual high-volume centres that repeatedly document survival rates surpassing 40% [5, 17, 18, 25, 26]. This volume to outcome relationship is well-established for both ECMO management [27] and the Norwood procedure itself [28]. Consequently, larger institutions typically manage higher volume of both operations and ECMO cases, likely contributing to their superior results and explaining the variability seen across smaller series and larger registries.

The risk factors associated with mortality in paediatric patients requiring ECMO support are well-documented across multiple studies. A large analysis by Sherwin et al. reported that these factors include the use of ECMO for failure to wean from cardiopulmonary bypass, the need for mechanical ventilation prior to ECMO initiation, extended duration of ECMO support, and the development of renal failure or neurologic injury [6]. These reported findings are consistent with other major studies, which identified prematurity and low weight as additional predictors of in-hospital mortality [5–7, 18, 29]. The pathogenesis of renal and neurologic complications in this group of patients is plausibly a result of low cardiac output state preceding cannulation or suboptimal organ perfusion following ECMO initiation. Therefore, the timely adoption of ECMO support, before the onset of irreversible organ damage as a result of poor perfusion, is paramount [23, 30]. Retrograde flow during ECMO support increases systemic afterload, thereby augmenting the pressure load encountered by the systemic atrioventricular valve. This may increase ventricular wall tension and myocardial oxygen demand and has been implicated in the development or worsening of atrioventricular valve regurgitation (AVVR) [31]. However, in our pooled analysis of four studies including 1,281 patients, the incidence of AVVR following ECMO after the Norwood procedure was comparable between groups, despite the well-documented haemodynamic burden associated with ECMO initiation.

Of note, few studies have specifically assessed the impact of postoperative ECMO timing on outcomes after paediatric cardiac surgery, none have focussed exclusively on patients undergoing the Norwood operation [32]. The existing evidence presents a complex picture. A large multicentre analysis of 2,908 children who received post-cardiotomy ECMO found that early cannulation (within one day of surgery) did not confer a survival benefit, though it was associated with reduced intensive care unit and hospital lengths of stay [33]. This finding of no mortality difference based on timing is corroborated by another large study over 2,000 patients [34]. On the other hand, Gupta et al. reported that each one-day delay in initiating ECMO was associated with small (1–3%), yet statistically significant, increases in ECMO duration, ventilation time, and ICU and hospital stay; however, these increases were considered clinically insignificant, suggesting that the clinical impact of delayed ECMO initiation in their cohort might be limited [33].

Despite the lack of clear survival advantage in these studies, the mechanisms underlying potential benefits remain to be fully outlined and may be influenced by variations in clinical indications and patient-specific factors. It is plausible that the early institution of ECMO provides a more stable platform for clinicians to promptly identify hemodynamically significant residual lesions, thereby enabling timely reintervention [24]. This concept is supported by an earlier study in which 83% of neonates requiring postcardiotomy ECMO had residual lesions, and a shorter time to their diagnosis or correction was significantly associated with survival [35]. Other potential reasons for a possible survival advantage include, earlier ECMO cannulation avoids prolonged hemodynamic instability or extensive medical management prior to ECMO, including earlier diagnoses of anatomical residua. The decision to cannulate earlier must be carefully balanced with the risks associated with ECMO including local thrombosis, emboli, or bleeding complications from anticoagulation [36].

Extensive ECMO duration is an important marker of failed myocardial recovery and is strongly associated with increased mortality. Pathophysiologically, ECMO activates a diffuse inflammatory process that can lead to subsequent immune suppression, heightened susceptibility to sepsis, and multi-organ dysfunction. Therefore, prolonged ECMO runs have been linked to late deaths from complications arising from these mechanistic sequelae after decannulation [26]. Consequently, it is important to minimise ECMO duration, including the early detection and management of any residual lesion that would otherwise impact weaning success.

Interestingly, consistent with our findings of comparable survival to stages 2 and 3 between patients who underwent ECMO and those that did not, improved survival rates have been reported in literature. For example, mid-term data on post-Norwood ECMO patients’ survival through subsequent surgical stages has been reported in literature. Debrunner et al. reported that 10 patients survived to hospital discharge; of these, 7 subsequently underwent a BCPC, and 4 ultimately survived to Fontan completion [18]. Likewise, Ravishanker et al. documented that 14 patients were early survivors, with 5 of those surviving to Fontan Completion [7]. In contrast, data published by Friedland-little et al. outlined more promising results, with higher survival rates to hospital discharge (44%), to BCPC (36%), and to Fontan completion (25%) [5].

These favourable results may reflect institutional variations in ECMO deployment, postoperative management and the rigor of post-discharge follow-up protocols. Variations in institutional protocols, particularly the constant improvements in paediatric cardiac critical care, which include the establishment of dedicated cardiac ICU [37, 38], the expansion of multidisciplinary care models, enhanced protocols for preventing hospital-acquired infections and postoperative complications (e.g., coagulopathy, low cardiac output syndrome), a refined understanding of anticoagulation management on ECMO, and the accumulated experience of the entire clinical team [39–43]. Finally, comprehensive parent education and meticulous post-discharge monitoring and follow-up are critical components of care that have been shown to improve overall survival and reduce interstage mortality. This is especially vital for high-risk patients to facilitate the early detection and management of life-threatening problems [10].

Furthermore, our pooled analysis of operative outcomes revealed that CPB time was significantly longer in the ECMO cohort, compared to a comparable cross-clamp and HCA times. This prolonged CPB duration is a key factor correlated with the significantly extended hospital and ICU recover periods in patients who required postoperative ECMO support [44], a trend also observed in our pooled analysis. Prolonged CPB times, vasoactive-inotrope score (VIS) and inhaled nitric oxide (iNO) requirement have been previously documented in literature as potential markers for ECMO requirement postoperatively. CPB time, VIS and iNO requirement can be targeted as modifiable risk factors for quality improvement initiatives [44–46]. Addressing these factors is a potential way to reduce the number of patients requirement ECMO after the Norwood operation, thereby shortening recovery times and improving survival. Consistent with our findings, several studies have reported no clear association between cross-clamp duration (minutes) and the outcome of ECMO post congenital cardiac surgery. For example, Khorsandi et al. found reduced survival when CPB duration exceeded 400 min and cross-clamp duration exceeded 120 min; however, these associations were not statistically significant on univariable analysis [47]. Similarly, Botan et al. reported no significant difference in cross-clamp time between survivors and non-survivors following paediatric cardiac surgery requiring ECMO, in a 7 year retrospective study [48].

Although the exact mechanism pertaining to the relationship between CPB time and postoperative ECMO is unclear, we speculate that prolonged CPB time is likely a multifactorial marker, reflecting both intrinsic anatomic complexity and potentially modifiable factors such as surgical technique and volume. This is supported by prior studies that have correlated surgeon and centre volume with morbidity and mortality after the Norwood procedure [7, 49, 50], this suggests that if surgeon experience influences CPB time, it may indirectly modify the risk of requiring postoperative ECMO. However, comparable HCA and cross-clamp times reported in our pooled analysis suggest that the challenge leading to longer CPB time is not related to the arch reconstruction. Ultimately, a longer CPB time may delay myocardial recovery, predisposing patients to a longer ECMO duration, longer postoperative recovery and stay and thus negatively impacting survival to subsequent stages of single ventricle repair. In addition to impact CPB times, anatomical complexity has been speculated to have implications on ECMO survival post Norwood operation.

**Limitations**

This meta-analysis is limited by the retrospective nature of the available evidence, which consisted predominantly of small, single-centre studies, thereby reducing generalisability and limiting statistical power to detect significant between-group differences. Additional clinical heterogeneity likely arose from institutional variation in ECMO cannulation thresholds, patient selection, indications for support, and evolving perioperative management practices over time. This is particularly important as ECMO is not a homogeneous intervention, and outcomes are likely influenced by the underlying indication for support (e.g., failure to wean from cardiopulmonary bypass versus cardiac arrest or postoperative haemodynamic instability). However, most included studies did not report outcomes stratified by ECMO indication, limiting more granular interpretation of treatment effect. Methodological heterogeneity was also introduced by inconsistencies in the inclusion of ECMO non-survivors, definitions of ICU duration, and follow-up periods, which likely contributed to heterogeneity in pooled analyses of Glenn completion, ICU stay, and unplanned reoperation. Furthermore, important clinically relevant variables, including ECMO indication, shunt morphology (Sano versus modified Blalock–Taussig shunt), and perioperative risk factors, were insufficiently reported to permit meaningful subgroup analyses. Finally, assessment of ECMO-related morbidity is susceptible to survivor bias, as the most severe complications may have contributed to early mortality and therefore be underrepresented in longer-term outcomes.

## Conclusion

Our analysis supports that ECMO use post-Norwood procedure identifies an high-risk subgroup with significantly higher early-mortality and resource utilization. However, this association must be interpreted cautiously due to heterogenous ECMO indications. Future efforts should focus on identifying modifiable risk factors to improve outcomes.

## Electronic Supplementary Material

Below is the link to the electronic supplementary material.

Supplementary Material 1

## Data Availability

No datasets were generated or analysed during the current study.

## Abbreviations

AA: Aortic atresia
AS: Aortic stenosis
AVVR: Atrioventricular valve regurgitation
BCPC: Bidirectional cavopulmonary connection (Glenn procedure)
CI: Confidence interval
CPB: Cardiopulmonary bypass
CPR: Cardiopulmonary resuscitation
CVVH: Continuous veno-venous hemofiltration
ECLS: Extracorporeal life support
ECMO: Extracorporeal membrane oxygenation
ECPR: Extracorporeal cardiopulmonary resuscitation (E-CPR)
HLHS: Hypoplastic left heart syndrome
ICU: Intensive care unit
iNO: Inhaled nitric oxide
MA: Mitral atresia
MD: Mean difference
OR: Odds ratio
PRISMA: Preferred reporting items for systematic reviews and meta-analyses
RCT: Randomized controlled trial
RR: Risk ratio
ROB2: Risk of Bias 2 (tool for randomized trials)
ROBINS-I: Risk of bias in non-randomized studies - of interventions
VA-ECMO: Venoarterial extracorporeal membrane oxygenation

## References

[1] Newburger JW, Sleeper LA, Gaynor JW, Hollenbeck-Pringle D, Frommelt PC, Li JS, et al. Transplant-Free Survival and Interventions at 6 Years in the SVR Trial. Circulation. 2018;137(21):2246–53. 10.1161/CIRCULATIONAHA.117.029375. 29437119 10.1161/CIRCULATIONAHA.117.029375 PMC5963989

[2] Mayr B, Kido T, Holder S, Wallner M, Vodiskar J, Strbad M, et al. Single-centre outcome of extracorporeal membrane oxygenation after the neonatal Norwood procedure. Eur J Cardiothorac Surg. 2022;62(3):ezac129. 10.1093/ejcts/ezac129. 35304610 10.1093/ejcts/ezac129

[3] Stasik CN, Goldberg CS, Bove EL, Devaney EJ, Ohye RG. Current outcomes and risk factors for the Norwood procedure. J Thorac Cardiovasc Surg. 2006;131(2):412–7. 10.1016/j.jtcvs.2005.09.030. 16434272 10.1016/j.jtcvs.2005.09.030

[4] Tweddell JS, Hoffman GM, Mussatto KA, Fedderly RT, Berger S, Jaquiss RDB, et al. Improved survival of patients undergoing palliation of hypoplastic left heart syndrome: lessons learned from 115 consecutive patients. Circulation. 2002;106(12 Suppl 1):I82–89. PubMed PMID: 12354714. 12354714

[5] Friedland-Little JM, Aiyagari R, Yu S, Donohue JE, Hirsch-Romano JC. Survival Through Staged Palliation: Fate of Infants Supported by Extracorporeal Membrane Oxygenation After the Norwood Operation. Ann Thorac Surg. 2014;97(2):659–65. 10.1016/j.athoracsur.2013.10.066. 24365213 10.1016/j.athoracsur.2013.10.066

[6] Sherwin ED, Gauvreau K, Scheurer MA, Rycus PT, Salvin JW, Almodovar MC, et al. Extracorporeal membrane oxygenation after stage 1 palliation for hypoplastic left heart syndrome. J Thorac Cardiovasc Surg. 2012;144(6):1337–43. 10.1016/j.jtcvs.2012.03.035. 22503203 10.1016/j.jtcvs.2012.03.035

[7] Ravishankar C, Dominguez TE, Kreutzer J, Wernovsky G, Marino BS, Godinez R, et al. Extracorporeal membrane oxygenation after stage I reconstruction for hypoplastic left heart syndrome*. Pediatr Crit Care Med. 2006;7(4):319–23. 10.1097/01.PCC.0000227109.82323.CE. 16738497 10.1097/01.PCC.0000227109.82323.CE

[8] Page MJ, McKenzie JE, Bossuyt PM, Boutron I, Hoffmann TC, Mulrow CD, et al. The PRISMA 2020 statement: an updated guideline for reporting systematic reviews. BMJ. 2021;n71. 10.1136/bmj.n71. 10.1136/bmj.n71 PMC8005924 33782057

[9] Sterne JA, Hernán MA, Reeves BC, Savović J, Berkman ND, Viswanathan M, et al. ROBINS-I: a tool for assessing risk of bias in non-randomised studies of interventions. BMJ. 2016. 10.1136/bmj.i4919. 27733354 10.1136/bmj.i4919 PMC5062054

[10] Alsoufi B, Wolf M, Botha P, Kogon B, McCracken C, Ehrlich A, et al. Late Outcomes of Infants Supported by Extracorporeal Membrane Oxygenation Following the Norwood Operation. World J Pediatr Congenit Heart Surg. 2015;6(1):9–17. 10.1177/2150135114558072. 25548338 10.1177/2150135114558072

[11] Beshish AG, Amedi A, Harriott A, Patel S, Evans S, Scheel A, et al. Short-term outcomes, functional status, and risk factors for requiring extracorporeal life support after Norwood operation: a single-center retrospective study. ASAIO J. 2024;70(4):328–35. 10.1097/MAT.0000000000002109. 38557688 10.1097/MAT.0000000000002109

[12] De Jesus-Brugman N, Hobson MJ, Herrmann JL, Friedman ML, Cordes T, Mastropietro CW. Improved outcomes in neonates who require venoarterial extracorporeal membrane oxygenation after the Norwood procedure. Int J Artif Organs. 2020;43(3):180–8. 10.1177/0391398819882020. 31623516 10.1177/0391398819882020

[13] Fernandez RP, Joy BF, Allen R, Stewart J, Miller-Tate H, Miao Y, et al. Interstage survival for patients with hypoplastic left heart syndrome after ECMO. Pediatr Cardiol. 2017;38(1):50–5. 10.1007/s00246-016-1483-7. 27803957 10.1007/s00246-016-1483-7

[14] Friedland-Little JM, Hirsch-Romano JC, Yu S, Donohue JE, Canada CE, Soraya P, et al. Risk factors for requiring extracorporeal membrane oxygenation support after a Norwood operation. J Thorac Cardiovasc Surg. 2014;148(1):266–72. 10.1016/j.jtcvs.2013.08.051. 24100094 10.1016/j.jtcvs.2013.08.051

[15] Friedland-Little JM, Uzark K, Yu S, Lowery R, Aiyagari R, Hirsch-Romano JC. Functional status and quality of life in survivors of extracorporeal membrane oxygenation after the Norwood operation. Ann Thorac Surg. 2017;103(6):1950–5. 10.1016/j.athoracsur.2016.11.015. 28223051 10.1016/j.athoracsur.2016.11.015

[16] Sengupta A, Gauvreau K, Kaza A, Allan C, Thiagarajan R, Del Nido PJ, et al. Influence of intraoperative residual lesions and timing of extracorporeal membrane oxygenation on outcomes following first-stage palliation of single-ventricle heart disease. J Thorac Cardiovasc Surg. 2023;165(6):2181-e21922. 10.1016/j.jtcvs.2022.06.028. 36058745 10.1016/j.jtcvs.2022.06.028

[17] Pizarro C, Davis DA, Healy RM, Kerins PJ, Norwood WI. Is there a role for extracorporeal life support after stage I Norwood? Eur J Cardiothorac Surg. 2001;19(3):294–301. 10.1016/S1010-7940(01)00575-9. 11251269 10.1016/s1010-7940(01)00575-9

[18] DeBrunner MG, Porayette P, Breinholt JP, Turrentine MW, Cordes TM. Midterm survival of infants requiring postoperative extracorporeal membrane oxygenation after Norwood palliation. Pediatr Cardiol. 2013;34(3):570–5. 10.1007/s00246-012-0499-x. 23007923 10.1007/s00246-012-0499-x

[19] Alsoufi B, Al-Radi OO, Gruenwald C, Lean L, Williams WG, McCrindle BW, et al. Extra-corporeal life support following cardiac surgery in children: analysis of risk factors and survival in a single institution. Eur J Cardiothorac Surg. 2009;35(6):1004–11. 10.1016/j.ejcts.2009.02.015. 19356943 10.1016/j.ejcts.2009.02.015

[20] Morris MC, Ittenbach RF, Godinez RI, Portnoy JD, Tabbutt S, Hanna BD, et al. Risk factors for mortality in 137 pediatric cardiac intensive care unit patients managed with extracorporeal membrane oxygenation*. Crit Care Med. 2004;32(4):1061–9. 10.1097/01.CCM.0000119425.04364.CF. 15071402 10.1097/01.ccm.0000119425.04364.cf

[21] Tabbutt S, Ghanayem N, Ravishankar C, Sleeper LA, Cooper DS, Frank DU, et al. Risk factors for hospital morbidity and mortality after the Norwood procedure: A report from the Pediatric Heart Network Single Ventricle Reconstruction trial. J Thorac Cardiovasc Surg. 2012;144(4):882–95. 10.1016/j.jtcvs.2012.05.019. 22704284 10.1016/j.jtcvs.2012.05.019 PMC4385520

[22] Saiki H, Kuwata S, Kurishima C, Masutani S, Senzaki H. Vulnerability of Coronary Circulation After Norwood Operation. Ann Thorac Surg. 2016;101(4):1544–51. 10.1016/j.athoracsur.2015.10.077. 26857638 10.1016/j.athoracsur.2015.10.077

[23] Allan CK, Thiagarajan RR, Del Nido PJ, Roth SJ, Almodovar MC, Laussen PC. Indication for initiation of mechanical circulatory support impacts survival of infants with shunted single-ventricle circulation supported with extracorporeal membrane oxygenation. J Thorac Cardiovasc Surg. 2007;133(3):660–7. 10.1016/j.jtcvs.2006.11.013. 17320562 10.1016/j.jtcvs.2006.11.013

[24] Ugaki S, Kasahara S, Kotani Y, Nakakura M, Douguchi T, Itoh H, et al. Extracorporeal Membrane Oxygenation Following Norwood Stage 1 Procedures at a Single Institution. Artif Organs. 2010;34(11):898–903. 10.1111/j.1525-1594.2010.01141.x. 21092032 10.1111/j.1525-1594.2010.01141.x

[25] Polimenakos AC, Wojtyla P, Smith PJ, Rizzo V, Nater M, El Zein CF, et al. Post-cardiotomy extracorporeal cardiopulmonary resuscitation in neonates with complex single ventricle: analysis of outcomes. Eur J Cardiothorac Surg. 2011;S1010794011002867. 10.1016/j.ejcts.2011.01.087. 10.1016/j.ejcts.2011.01.087 21507672

[26] Alsoufi B, Awan A, Manlhiot C, Al-Halees Z, Al-Ahmadi M, McCrindle BW, et al. Does Single Ventricle Physiology Affect Survival of Children Requiring Extracorporeal Membrane Oxygenation Support Following Cardiac Surgery? World J Pediatr Congenit Heart Surg. 2014;5(1):7–15. 10.1177/2150135113507292. 24403349 10.1177/2150135113507292

[27] Karamlou T, Vafaeezadeh M, Parrish AM, Cohen GA, Welke KF, Permut L, et al. Increased extracorporeal membrane oxygenation center case volume is associated with improved extracorporeal membrane oxygenation survival among pediatric patients. J Thorac Cardiovasc Surg. 2013;145(2):470–5. 10.1016/j.jtcvs.2012.11.037. 23246046 10.1016/j.jtcvs.2012.11.037

[28] Hornik CP, He X, Jacobs JP, Li JS, Jaquiss RDB, Jacobs ML, et al. Relative Impact of Surgeon and Center Volume on Early Mortality After the Norwood Operation. Ann Thorac Surg. 2012;93(6):1992–7. 10.1016/j.athoracsur.2012.01.107. 22516833 10.1016/j.athoracsur.2012.01.107 PMC3469698

[29] Jolley M, Yarlagadda VV, Rajagopal SK, Almodovar MC, Rycus PT, Thiagarajan RR. Extracorporeal Membrane Oxygenation–Supported Cardiopulmonary Resuscitation Following Stage 1 Palliation for Hypoplastic Left Heart Syndrome*. Pediatr Crit Care Med. 2014;15(6):538–45. 10.1097/PCC.0000000000000159. 24797720 10.1097/PCC.0000000000000159

[30] Jaggers JJ, Forbess JM, Shah AS, Meliones JN, Kirshbom PM, Miller CE, et al. Extracorporeal membrane oxygenation for infant postcardiotomy support: significance of shunt management. Ann Thorac Surg. 2000;69(5):1476–83. 10.1016/S0003-4975(00)01330-8. 10881826 10.1016/s0003-4975(00)01330-8

[31] Werdan K, Gielen S, Ebelt H, Hochman JS. Mechanical circulatory support in cardiogenic shock. Eur Heart J. 2014;35(3):156–67. 10.1093/eurheartj/eht248. 24014384 10.1093/eurheartj/eht248

[32] Gupta P, DasGupta R, Best D, Chu CB, Elsalloukh H, Gossett JM, et al. Delayed extracorporeal membrane oxygenation in children after cardiac surgery: two-institution experience. Cardiol Young. 2015;25(2):248–54. 10.1017/S1047951113002011. 24345676 10.1017/S1047951113002011

[33] Gupta P, Robertson MJ, Rettiganti M, Seib PM, Wernovsky G, Markovitz BP, et al. Impact of Timing of ECMO Initiation on Outcomes After Pediatric Heart Surgery: A Multi-Institutional Analysis. Pediatr Cardiol. 2016;37(5):971–8. 10.1007/s00246-016-1379-6. 27037549 10.1007/s00246-016-1379-6

[34] Agarwal HS, Hardison DC, Saville BR, Donahue BS, Lamb FS, Bichell DP, et al. Residual lesions in postoperative pediatric cardiac surgery patients receiving extracorporeal membrane oxygenation support. J Thorac Cardiovasc Surg. 2014;147(1):434–41. 10.1016/j.jtcvs.2013.03.021. 23597724 10.1016/j.jtcvs.2013.03.021

[35] Howard TS, Kalish BT, Wigmore D, Nathan M, Kulik TJ, Kaza AK, et al. Association of Extracorporeal Membrane Oxygenation Support Adequacy and Residual Lesions With Outcomes in Neonates Supported After Cardiac Surgery*. Pediatr Crit Care Med. 2016;17(11):1045–54. 10.1097/PCC.0000000000000943. 27648896 10.1097/PCC.0000000000000943

[36] Seeber F, Krenner N, Sames-Dolzer E, Tulzer A, Srivastava I, Kreuzer M, et al. Outcome after extracorporeal membrane oxygenation therapy in Norwood patients before the bidirectional Glenn operation. Eur J Cardiothorac Surg. 2024;65(4):ezae153. 10.1093/ejcts/ezae153. 38603622 10.1093/ejcts/ezae153

[37] Eldadah M, Leo S, Kovach K, Ricardo Argueta Morales I, Pepe J, Fakioglu H, et al. Influence of a dedicated paediatric cardiac intensive care unit on patient outcomes. Nurs Crit Care. 2011;16(6):281–6. 10.1111/j.1478-5153.2011.00455.x. 21999418 10.1111/j.1478-5153.2011.00455.x

[38] Festa M, Winlaw DS. Dedicated cardiac intensive care units: Good for the patient, good for the surgeon. J Thorac Cardiovasc Surg. 2018;155(6):2604–5. 10.1016/j.jtcvs.2018.02.041. 29588081 10.1016/j.jtcvs.2018.02.041

[39] Barker GM, O’Brien SM, Welke KF, Jacobs ML, Jacobs JP, Benjamin DK, et al. Major Infection After Pediatric Cardiac Surgery: A Risk Estimation Model. Ann Thorac Surg. 2010;89(3):843–50. 10.1016/j.athoracsur.2009.11.048. 20172141 10.1016/j.athoracsur.2009.11.048 PMC3319976

[40] Caruso TJ, Wang EY, Schwenk H, Marquez JLS, Cahn J, Loh L, et al. A Postoperative Care Bundle Reduces Surgical Site Infections in Pediatric Patients Undergoing Cardiac Surgeries. Jt Comm J Qual Patient Saf. 2019;45(3):156–63. 10.1016/j.jcjq.2018.05.009. 30170753 10.1016/j.jcjq.2018.05.009

[41] Gaies MG, Clarke NS, Donohue JE, Gurney JG, Charpie JR, Hirsch JC. Personnel and unit factors impacting outcome after cardiac arrest in a dedicated pediatric cardiac intensive care unit*. Pediatr Crit Care Med. 2012;13(5):583–8. 10.1097/PCC.0b013e318238b272. 22079956 10.1097/PCC.0b013e318238b272

[42] Hickey PA, Pasquali SK, Gaynor JW, He X, Hill KD, Connor JA, et al. Critical Care Nursing’s Impact on Pediatric Patient Outcomes. Ann Thorac Surg. 2016;102(4):1375–80. 10.1016/j.athoracsur.2016.03.019. 27173065 10.1016/j.athoracsur.2016.03.019

[43] Donnelly JP, Raffel DM, Shulkin BL, Corbett JR, Bove EL, Mosca RS, et al. Resting coronary flow and coronary flow reserve in human infants after repair or palliation of congenital heart defects as measured by positron emission tomography. J Thorac Cardiovasc Surg. 1998;115(1):103–10. 10.1016/S0022-5223(98)70448-9. 9451052 10.1016/s0022-5223(98)70448-9

[44] Aladwan H, Alshoubaki W, Alqaisi AI, Abuamereh HA, Alatoum LM, Mohd AF. Impact of duration of cardiopulmonary bypass on recovery after open heart surgery. Int J Adv Med. 2024;11(3):185–8. 10.18203/2349-3933.ijam20240922.

[45] For the Western Canadian Complex Pediatric Therapies Follow-up Program, Kuraim GA, Garros D, Ryerson L, Moradi F, Dinu IA, et al. Predictors and outcomes of early post-operative veno-arterial extracorporeal membrane oxygenation following infant cardiac surgery. j intensive care. 2018;6(1):56. 10.1186/s40560-018-0326-4. 10.1186/s40560-018-0326-4 PMC6122608 30202528

[46] Yates AR, Berger JT, Reeder RW, Banks R, Mourani PM, Berg RA, et al. Characterization of Inhaled Nitric Oxide Use for Cardiac Indications in Pediatric Patients*. Pediatr Crit Care Med. 2022;23(4):245–54. 10.1097/PCC.0000000000002917. 35200229 10.1097/PCC.0000000000002917 PMC9058189

[47] Khorsandi M, Davidson M, Bouamra O, McLean A, MacArthur K, Torrance I, et al. Extracorporeal membrane oxygenation in pediatric cardiac surgery: A retrospective review of trends and outcomes in Scotland. Annals of Pediatric Cardiology. 2018;11(1):3. 10.4103/apc.APC_88_17. 29440824 10.4103/apc.APC_88_17 PMC5803974

[48] Botan E, Aslan AD, Gün E, Havan M, Dikmen N, Gurbanov A, et al. Extracorporeal Membrane Oxygenation after Pediatric Cardiac Surgery: A Single-Center Experience. Turk Arch Pediatr. 2024;59(4):358–63. 10.5152/TurkArchPediatr.2024.23291. 39140771 10.5152/TurkArchPediatr.2024.23291 PMC11332433

[49] Mahle WT, Spray TL, Wernovsky G, Gaynor JW, Clark BJ. Survival After Reconstructive Surgery for Hypoplastic Left Heart Syndrome: A 15-Year Experience From a Single Institution. Circulation. 2000;102(Supplement 3):III–136. 10.1161/01.CIR.102.suppl_3.III-136. 10.1161/01.cir.102.suppl_3.iii-136 11082376

[50] Thiagarajan RR, Laussen PC, Rycus PT, Bartlett RH, Bratton SL. Extracorporeal Membrane Oxygenation to Aid Cardiopulmonary Resuscitation in Infants and Children. Circulation. 2007;116(15):1693–700. 10.1161/CIRCULATIONAHA.106.680678. 17893278 10.1161/CIRCULATIONAHA.106.680678

