# Supplementary material for: Outcomes after extracorporeal membrane oxygenation following the norwood procedure: a systematic review and meta-analysis
Source: J Cardiothorac Surg. 2026 May 23;21:514. doi: 10.1186/s13019-026-04232-4 (PMC13449676; doi:10.1186/s13019-026-04232-4)
Supplement: Supplementary file 1 [file 13019_2026_4232_MOESM1_ESM.docx]

**Supplementary Material**

**Supplementary Table 1.** MEDLINE search strategy

**Supplementary Table 2.** ROBINS-1 Risk of Bias assessment

**Supplementary Figure 1.** Funnel plot analysis of risk of publication bias (primary outcome: short-term mortality).

**Supplementary Figure 2.** Sensitivity Analysis Forest plot for Glenn completion. ECMO= Extracorporeal Membrane Oxygenation, MD= Mean Difference, OR= odds ratio, RR= risk ratio, CI= confidence interval.

**Supplementary Figure 3.** Sensitivity Analysis Forest plot for cross-clamp time (miutes). ECMO= Extracorporeal Membrane Oxygenation, MD= Mean Difference, OR= odds ratio, RR= risk ratio, CI= confidence interval.

**Supplementary Figure 4.** Sensitivity Analysis Forest plot for hospital length of stay (days). ECMO= Extracorporeal Membrane Oxygenation, MD= Mean Difference, OR= odds ratio, RR= risk ratio, CI= confidence interval.

**Supplementary Figure 5.** Sensitivity Analysis Forest plot for ICU length of stay (days). ECMO= Extracorporeal Membrane Oxygenation, MD= Mean Difference, OR= odds ratio, RR= risk ratio, CI= confidence interval.

**Supplementary Figure 6.** Forest plot for unplanned reoperation. ECMO= Extracorporeal Membrane Oxygenation, MD= Mean Difference, OR= odds ratio, RR= risk ratio, CI= confidence interval.

**Supplementary Figure 7.** Sensitivity analysis forest plot for unplanned reoperation. ECMO= Extracorporeal Membrane Oxygenation, MD= Mean Difference, OR= odds ratio, RR= risk ratio, CI= confidence interval.

**Supplementary Figure 8.** Forest plot for interstage death or transplantation. ECMO= Extracorporeal Membrane Oxygenation, MD= Mean Difference, OR= odds ratio, RR= risk ratio, CI= confidence interval.

**Supplementary Figure 9.** Sensitivity analysis forest plot for interstage death or transplantation. ECMO= Extracorporeal Membrane Oxygenation, MD= Mean Difference, OR= odds ratio, RR= risk ratio, CI= confidence interval.

**Supplementary Figure 10.** Forest plot for incidence of atrioventricular valve regurgitation. ECMO= Extracorporeal Membrane Oxygenation, MD= Mean Difference, OR= odds ratio, RR= risk ratio, CI= confidence interval.

**Supplementary Figure 11.** A Meta-Regression plot for interstage death of transplantation based on median year of publication.

**Supplementary Figure 12.** A Meta-Regression plot for survival to Glenn operation based on median year of publication.

**Supplementary Figure 13.** A Meta-Regression plot for unplanned reoperation based on median year of publication.

**Supplementary Figure 14.** A Meta-Regression plot for cross-clamp time (minutes) based on median year of publication.

**Supplementary Figure 15.** A Meta-Regression plot for ICU length of stay based on median year of publication.

**Supplementary Figure 16.** A Meta-Regression plot for Hospital length of stay based on median year of publication.

**Supplementary Table 1.** Complete Search Strategy and PubMed Search Strategy

Complete Search Strategy

PubMed: 1703

Web of Science: 1939

Cochrane: 62

EMBASE: 3658

Total studies with duplicates: 7362

Total studies without duplicates: 5162

**Search Strategy for Ovid MEDLINE.**

**Search: ("Single ventricle" OR "hypoplastic left heart syndrome" OR "HLHS" OR "functionally univentricular heart" OR "univentricular circulation" OR "univentricular physiology" OR "congenital single ventricle" OR "Norwood procedure" OR "Norwood stage 1" OR "Norwood palliation" OR "stage 1 palliation" OR "first-stage palliation" OR "first-stage Norwood" OR "Fontan procedure" OR "Fontan circulation" OR "Fontan-associated" OR "Fontan failure" OR "congenital heart disease" OR "critical congenital heart disease" OR "CHD" OR "cyanotic congenital heart disease" OR "complex congenital heart disease" OR "pediatric heart surgery") AND ("ECMO" OR "extracorporeal membrane oxygenation" OR "extracorporeal life support" OR "ECLS" OR "mechanical circulatory support" OR "MCS" OR "VA-ECMO" OR "veno-arterial ECMO" OR "cardiac ECMO" OR "cardiac extracorporeal support" OR "extracorporeal cardiopulmonary support" OR "neonatal ECMO" OR "pediatric ECMO" OR "heart-lung support" OR "ECMO therapy" OR "ECMO management" OR "postoperative extracorporeal support" OR "rescue ECMO" OR "emergency ECMO" OR "ECMO outcomes" OR "ECMO survival" OR "cardiac arrest ECMO" OR "ECMO-assisted resuscitation" OR "ECMO complications" OR "ECPR")**

**Translations**

("Single ventricle"[All Fields] OR "hypoplastic left heart syndrome"[All Fields] OR "HLHS"[All Fields] OR "functionally univentricular heart"[All Fields] OR "univentricular circulation"[All Fields] OR "univentricular physiology"[All Fields] OR "congenital single ventricle"[All Fields] OR "Norwood procedure"[All Fields] OR "Norwood stage 1"[All Fields] OR "Norwood palliation"[All Fields] OR "stage 1 palliation"[All Fields] OR "first-stage palliation"[All Fields] OR "first-stage Norwood"[All Fields] OR "Fontan procedure"[All Fields] OR "Fontan circulation"[All Fields] OR "Fontan-associated"[All Fields] OR "Fontan failure"[All Fields] OR "congenital heart disease"[All Fields] OR "critical congenital heart disease"[All Fields] OR "CHD"[All Fields] OR "cyanotic congenital heart disease"[All Fields] OR "complex congenital heart disease"[All Fields] OR "pediatric heart surgery"[All Fields]) AND ("ECMO"[All Fields] OR "extracorporeal membrane oxygenation"[All Fields] OR "extracorporeal life support"[All Fields] OR "ECLS"[All Fields] OR "mechanical circulatory support"[All Fields] OR "MCS"[All Fields] OR "VA-ECMO"[All Fields] OR "veno-arterial ECMO"[All Fields] OR "cardiac ECMO"[All Fields] OR (("cardiacs"[All Fields] OR "heart"[MeSH Terms] OR "heart"[All Fields] OR "cardiac"[All Fields]) AND ("extracorporal"[All Fields] OR "extracorporally"[All Fields] OR "extracorporeal"[All Fields] OR "extracorporeally"[All Fields]) AND ("support"[All Fields] OR "support s"[All Fields] OR "supported"[All Fields] OR "supporter"[All Fields] OR "supporter s"[All Fields] OR "supporters"[All Fields] OR "supporting"[All Fields] OR "supportive"[All Fields] OR "supportiveness"[All Fields] OR "supports"[All Fields])) OR "extracorporeal cardiopulmonary support"[All Fields] OR "neonatal ECMO"[All Fields] OR "pediatric ECMO"[All Fields] OR "heart-lung support"[All Fields] OR "ECMO therapy"[All Fields] OR "ECMO management"[All Fields] OR "postoperative extracorporeal support"[All Fields] OR "rescue ECMO"[All Fields] OR "emergency ECMO"[All Fields] OR "ECMO outcomes"[All Fields] OR "ECMO survival"[All Fields] OR (("heart arrest"[MeSH Terms] OR ("heart"[All Fields] AND "arrest"[All Fields]) OR "heart arrest"[All Fields] OR ("cardiac"[All Fields] AND "arrest"[All Fields]) OR "cardiac arrest"[All Fields]) AND ("extracorporeal membrane oxygenation"[MeSH Terms] OR ("extracorporeal"[All Fields] AND "membrane"[All Fields] AND "oxygenation"[All Fields]) OR "extracorporeal membrane oxygenation"[All Fields] OR "ECMO"[All Fields])) OR ("ecmo assisted"[All Fields] AND ("resuscitability"[All Fields] OR "resuscitate"[All Fields] OR "resuscitated"[All Fields] OR "resuscitates"[All Fields] OR "resuscitating"[All Fields] OR "resuscitation"[MeSH Terms] OR "resuscitation"[All Fields] OR "resuscitations"[All Fields] OR "resuscitative"[All Fields] OR "resuscitator"[All Fields] OR "resuscitators"[All Fields])) OR "ECMO complications"[All Fields] OR "ECPR"[All Fields])

**Supplementary Table 2.** ROBINS-1 Risk of Bias assessment

**Supplementary Figure 1.** Funnel plot analysis of risk of publication bias (primary outcome: short-term mortality), and Egger’s test result.

**Supplementary Figure 2.** Sensitivity Analysis Forest plot for Glenn completion. ECMO= Extracorporeal Membrane Oxygenation, MD= Mean Difference, OR= odds ratio, RR= risk ratio, CI= confidence interval.

**Supplementary Figure 3.** Sensitivity Analysis Forest plot for cross-clamp time (minutes). ECMO= Extracorporeal Membrane Oxygenation, MD= Mean Difference, OR= odds ratio, RR= risk ratio, CI= confidence interval.

**Supplementary Figure 4.** Sensitivity Analysis Forest plot for hospital length of stay (days). ECMO= Extracorporeal Membrane Oxygenation, MD= Mean Difference, OR= odds ratio, RR= risk ratio, CI= confidence interval.

**Supplementary Figure 5.** Sensitivity Analysis Forest plot for ICU length of stay (days). ECMO= Extracorporeal Membrane Oxygenation, MD= Mean Difference, OR= odds ratio, RR= risk ratio, CI= confidence interval.

**Supplementary Figure 6.** Forest plot for unplanned reoperation. ECMO= Extracorporeal Membrane Oxygenation, MD= Mean Difference, OR= odds ratio, RR= risk ratio, CI= confidence interval.

**Supplementary Figure 7.** Sensitivity analysis forest plot for unplanned reoperation. ECMO= Extracorporeal Membrane Oxygenation, MD= Mean Difference, OR= odds ratio, RR= risk ratio, CI= confidence interval.

**Supplementary Figure 8.** Forest plot for interstage death or transplantation. ECMO= Extracorporeal Membrane Oxygenation, MD= Mean Difference, OR= odds ratio, RR= risk ratio, CI= confidence interval.

**Supplementary Figure 9.** Sensitivity analysis forest plot for interstage death or transplantation. ECMO= Extracorporeal Membrane Oxygenation, MD= Mean Difference, OR= odds ratio, RR= risk ratio, CI= confidence interval.

**Supplementary Figure 10.** Forest plot for incidence of atrioventricular valve regurgitation. ECMO= Extracorporeal Membrane Oxygenation, MD= Mean Difference, OR= odds ratio, RR= risk ratio, CI= confidence interval.

**Supplementary Figure 11.** A Meta-Regression plot for interstage death of transplantation based on median year of publication.

**Supplementary Figure 12.** A Meta-Regression plot for survival to Glenn operation based on median year of publication.

**Supplementary Figure 13.** A Meta-Regression plot for unplanned reoperation based on median year of publication.

**Supplementary Figure 14.** A Meta-Regression plot for cross-clamp time (minutes) based on median year of publication.

**Supplementary Figure 15.** A Meta-Regression plot for ICU length of stay based on median year of publication.

**Supplementary Figure 16.** A Meta-Regression plot for Hospital length of stay based on median year of publication.
